# Antinociceptive and Anti-Inflammatory Activities of Acetonic Extract from *Bougainvillea x buttiana* (var. Rose)

**DOI:** 10.3390/ph17081037

**Published:** 2024-08-06

**Authors:** Gabriela Castañeda-Corral, Mayra Cedillo-Cortezano, Magdalena Aviles-Flores, Misael López-Castillo, Juan José Acevedo-Fernández, Vera L. Petricevich

**Affiliations:** Facultad de Medicina, Universidad Autónoma del Estado de Morelos, Calle Iztaccihuatl Esq. Leñeros, Col. Volcanes, Cuernavaca 62350, Morelos, Mexico; gabriela.castaneda@uaem.mx (G.C.-C.); mayra.cedillo@uaem.edu.mx (M.C.-C.); magda.aviles0711@gmail.com (M.A.-F.); misael.pez@gmail.com (M.L.-C.); juan.acevedo@uaem.mx (J.J.A.-F.)

**Keywords:** acetonic extract *Bougainvillea x buttiana*, anti-nociceptive activity, anti-inflammatory activity, COX- and PLA2 inhibition, in silico analysis

## Abstract

**Background:***Bougainvillea x buttiana* is an ornamental plant with antioxidant, anti-inflammatory, and cytotoxic activities, which has been traditionally used to treat respiratory diseases. This study aimed to investigate whether the acetonic extract of *Bougainvillea x buttiana* var. Rose (BxbRAE-100%) has analgesic and anti-inflammatory properties and its potential action mechanisms. **Methods:** Analgesic and anti-inflammatory activities were evaluated using three murine pain models and two acute inflammation models. In vitro, the ability of the extract to inhibit proteolytic activity and the activities of the enzymes phospholipase A2 (PLA2) and cyclooxygenase (COX) were evaluated. In silico analysis was performed to predict the physicochemical and Absorption, distribution, metabolism, and excretion (ADME) profiles of the compounds previously identified in BxbRAE-100%. **Results:** In vivo BxbRAE-100% decreased the nociceptive behaviors in the writhing model, the tail immersion, and the formalin test, suggesting that the extract has the potential to relieve pain at peripheral and central levels. Additionally, topical or oral BxbRAE-100% treatment reduced dose-dependent 12-*O*-Tetradecanoylphorbol-13-acetate (TPA)-induced ear inflammation and carrageenan-induced paw edema, respectively. In vitro, BxbRAE-100% significantly inhibited proteolytic activity and PLA2, COX-1 and COX-2 activities. In silico, the compounds previously identified in BxbRAE-100% met Lipinski’s rule of five and showed adequate ADME properties. **Conclusions:** These results support the use of *B. x buttiana* in Traditional Mexican Medicine and highlight its potential for the development of new treatments for pain and inflammation.

## 1. Introduction

Inflammation is the response of the human body to a wound or infection. It is a fundamental mechanism for the clearance of the infectious agent and repairing the affected tissues. The goals of the inflammatory process are the complete recovery of health and return to homeostasis through cellular and molecular processes [1]. However, inflammation is also associated with both pain and multiple conditions, such as autoimmune diseases like arthritis and osteoarthritis, sports injuries, headaches, and musculoskeletal pain [2,3,4]. Pain is defined as an unpleasant sensory and emotional experience associated with, or resembling that associated with, actual or potential tissue damage [5]. Acute pain is a symptom that acts as a warning signal for the body to protect it from possible damage or injury and, thus, avoid harmful consequences. Therefore, it has an adaptive role. However, when pain becomes chronic, it can have detrimental effects on individuals’ health and quality of life, and sometimes, more than a symptom, it can be a disease by itself [6].

Standard anti-inflammatory and analgesic treatments include nonsteroidal anti-inflammatory drugs (NSAIDs) and glucocorticoids. However, these medications are associated with several side effects that affect the gastrointestinal and cardiovascular systems, kidneys, and liver, among other organs [7]. Furthermore, an insufficient response to treatment is frequently observed. As a result, the scientific community is seeking novel drugs and therapeutic agents for inflammation and pain management, particularly those of natural origin [8].

It is worth noting that medicinal plants are important sources of compounds with high therapeutic value. Examples include the approved drugs morphine, quinine, and acetyl-salicylic acid [9]. The discovery of these drugs has significantly contributed to the development of knowledge about chemical structures and mechanisms of action [10]. Furthermore, there are numerous reports in the literature about the presence of a wide variety of bioactive compounds found in medicinal plants, which exhibit beneficial biological activities such as analgesic, antibacterial, anti-inflammatory, antipyretic, antispasmodic, and antitumor activities [11]. Therefore, pure compounds or standardized extracts provide more opportunities to develop new medications. The World Health Organization aims to ensure the quality of plants for medicinal use. In this sense, the techniques to obtain extracts have been standardized because extraction, separation, and identification are critical steps that can potentially modify the type and number of bioactive compounds present in an extract [12].

*Bougainvillea* is a genus consisting of at least 18 species of ornamental and horticultural plants with colorful flowers belonging to the Nyctaginaceae family. It is endemic to Brazil and other South American regions. It was introduced pantropically and distributed in warm regions of Mexico, Asia, Australia, the Caribbean, South Africa, and the United States [13]. In Mexico, several species of *Bougainvillea* exist, including *B. spectabilis*, *B. glabra*, and *B. x buttiana*. *Bougainvillea* bracts, which are commonly confused with flower petals, are the most used part in traditional Mexican medicine to treat respiratory conditions such as cough, asthma, flu, and bronchitis. It has also been reported to be useful in treating gastrointestinal problems such as diarrhea and dysentery, as well as people suffering from lung pain, whooping cough, drowning, nausea, and wound cleaning [14]. It is commonly used in traditional medicine as an infusion of flowers and bracts that are administered orally [15].

The antinociceptive and anti-inflammatory properties of the ethanolic extract of *B. x buttiana* (var. Orange) were described for the first time by Alvarez Perez Gil et al., 2012 [16]. Subsequent studies found that the extract has significant antioxidant and immunomodulatory activities [17,18]. In our previous report, several extracts of *B. x buttiana* (var. Rose) of different polarities demonstrated cytotoxic and antioxidant activities. The phytochemical analysis showed that the polarity of the solvent influenced the composition of the extracts. In this sense, the main compounds identified in the extracts of *B. x buttiana* (var. Rose) include flavonoids (e.g., rutin and quercetin), carbohydrates (e.g., 3-*O*-Methyl-d-glucose), saturated fatty acids (e.g., *n*-Hexadecanoic acid) and polyunsaturated (e.g., 9,12-octadecadienoic (Z,Z)-), fatty alcohols (e.g., 1-Dotriacontanol), and phytosterols (e.g., Stigmasta-5,22-dien-3-ol and Stig-mast-7-en-3-ol, (3β,5α)) [19,20]. However, the antinociceptive and anti-inflammatory activities and mechanism of action of extracts made from flowers and bracts of plants of different colors have not yet been established.

This study aims to investigate the in vivo and in vitro anti-inflammatory activity of the acetonic extract of *Bougainvillea x buttiana* (var. Rose) (BxbRAE) as well as its peripheral and central analgesic actions. Finally, an in silico analysis of the physicochemical and ADME profiles of the bioactive compounds previously identified in the extract was performed. 

## 2. Results

### 2.1. Extraction and Yields

In this study, the extraction process was conducted with 8.38 g of flowers and bracts, in acetone (100%), and the yield obtained was 2.37%. The solid residue obtained was resuspended in 5% Tween 20 in saline solution and orally administered at different concentrations. The extraction yield was used as an indicator of the effects of extraction conditions. Under the test conditions used in this study, no mouse mortality or macroscopic changes in the skin, eyes, or fur were observed.

### 2.2. Analgesic Activity

**Figure 1:**
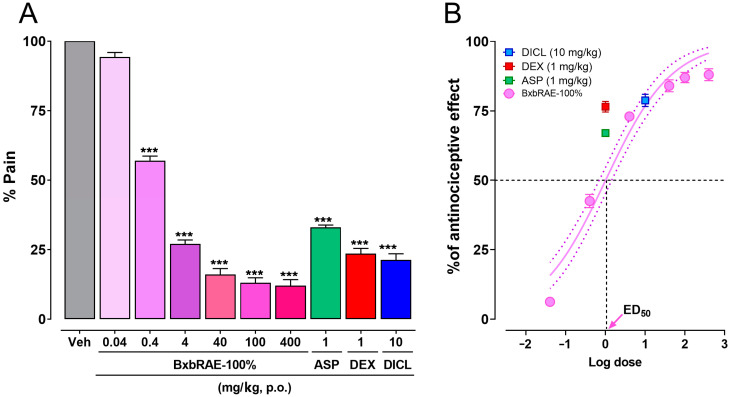
BxbRAE decreased in a dose-dependent manner acetic acid-induced pain in mice. (**A**) Bar graph showing the effect of BxbRAE 100% in the % of pain. (**B**) Dose–response curve (DRC) of the anti-nociceptive effect. In (**B**), the dashed lines represent the dose that achieves 50% of the anti-nociceptive effect (ED_50_). Aspirin (ASP), dexamethasone (DEX), and diclofenac (DICL) were used as positive controls. Data are shown as the mean ± SD (*n* = 4). *** *p* < 0.001 vs. the Veh group through one-way ANOVA followed by Dunnett’s test.

In this study, the antinociceptive properties of the acetonic extract from *Bougainvillea x buttiana* var. Rose (BxbRAE-100%) were evaluated using three different murine models: the acetic acid-induced writhing model, the tail immersion test, and the formalin test. Figure 1 shows the results obtained in the writhing model. It was observed that the groups of animals treated orally with BxbRAE 100% in the dose range between 0.4 and 400 mg/kg showed a statistically significant decrease in the percentage of pain (% pain) compared to the group treated with the vehicle. The doses of 40, 100, and 400 mg/kg showed the most significant reduction in % pain and were more effective than aspirin (1 mg/kg), dexamethasone (1 mg/kg), and diclofenac (10 mg/kg; Figure 1A). The dose-response curve analysis showed that the antinociceptive effect of BxbRAE 100% was dose-dependent, with a calculated ED_50_ of 10.14 mg/kg and an average maximum effect of 86 ± 2.0% (Figure 1B).

**Figure 2:**
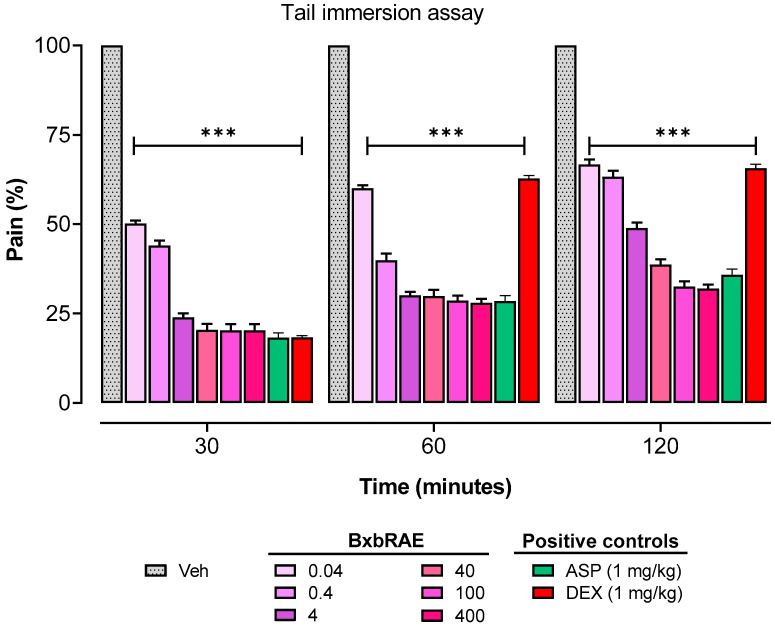
Effect of BxbRAE-100% in the tail immersion assay in mice. Aspirin (ASP) and dexamethasone (DEX) were used as positive controls. Data are shown as the mean ± SD (*n* = 4). *** *p* < 0.001 vs. the Veh group through one-way ANOVA followed by the Dunnett’s test.

The pain experimental design included the evaluation of the effect of the extract on pain induced by a thermal noxious stimulus using the tail immersion test. The pain percentages obtained from the tail immersion assay are presented in Figure 2. The results showed that all doses of the extract used in this study significantly reduced the pain percentage compared to the results of the group treated with the vehicle (*p* < 0.001). This reduction in the percentage of pain was observed from the first 30 min to 120 min of the test. The pain percentages in the BxbRAE-100%-treated groups were equal to those observed in the aspirin-treated group. In contrast, the pain percentages of the dexamethasone-treated group at 60 and 120 min were significantly higher than those of the BxbRAE-100% and aspirin.

**Figure 3:**
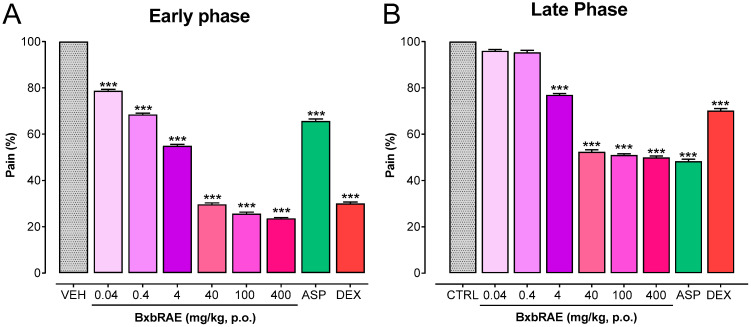
BxbRAE-100% decreased in a dose-dependent manner formalin-induced pain behaviors. Aspirin (ASP; 1 mg/kg) and dexamethasone (DEX; 1 mg/kg) were used as positive controls. Data are shown as the mean ± SD (*n* = 4). *** *p* < 0.001 vs. the Veh group through one-way ANOVA followed by the Dunnet’s test.

Formalin-induced pain behaviors is a model used to determine the central and peripheral activity of substances with analgesic properties, such as BxbRAE-100%. This test involves two phases: the early phase (0 to 5 min post-formalin injection) and the late phase (15 to 30 min post-formalin injection). In the present study, the results showed that the pretreatment of BxbRAE-100% at the doses between 0.04 and 400 mg/ kg significantly reduced pain percentages in a dose-dependent manner in both the early (Figure 3A) and the late phase (Figure 3B) of the formalin test. In both phases, the groups treated with the doses of 40, 100, and 400 mg/kg displayed the lowest pain percentages and were akin to those observed in the dexamethasone- or aspirin-treated groups in the early or late phases, respectively. 

### 2.3. In Vivo Anti-Inflammatory Activity

**Figure 4:**
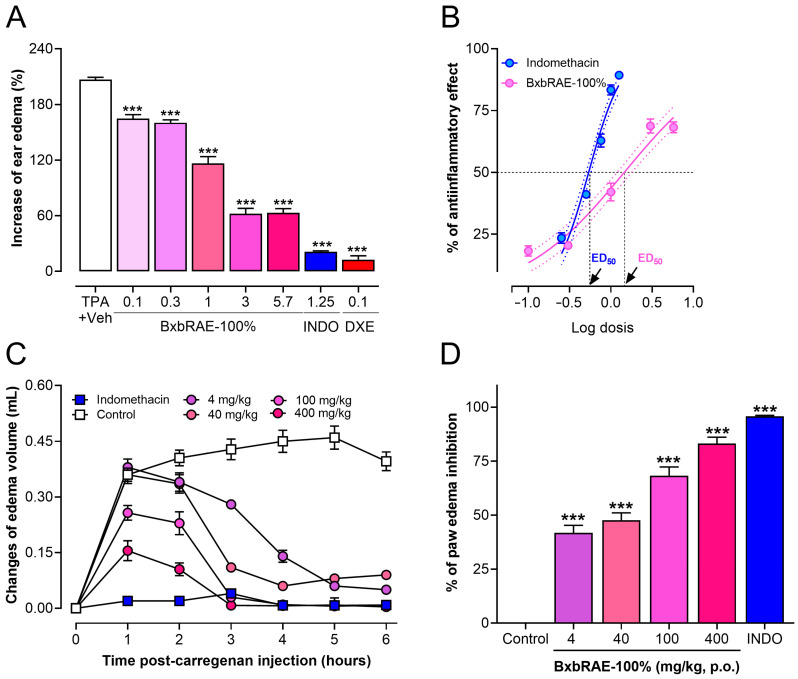
BxbRAE-100% reduced ear and paw edema in mice. (**A**) Effect of the topical treatment with the extract in the TPA-induced ear edema model. (**B**) Dose–response curve (DRC) of the anti-inflammatory effect of BxbRAE-100% vs. the positive control indomethacin (INDO). The dashed lines represent the dose that achieves a 50% anti-inflammatory effect (ED_50_). (**C**) Effect of BxbRAE-100% on the time course of carrageenan-induced paw edema. (**D**) Data are presented as the % of paw edema inhibition. Each point or bar represents the average ± SD. *** *p* < 0.001 vs. the Veh + TPA group or control group through one-way ANOVA followed by Dunnett’s test.

To determine the anti-inflammatory activity of BxbRAE-100% two murine models of inflammation were used. First, the topical anti-inflammatory activity of the extract was determined using the TPA (12-*O*-Tetradecanoylphorbol-13-acetate)-induced ear edema. Thus, increasing doses of BxbRAE-100% (0.1–0.57 mg/ear) or the positive controls indomethacin (0.25–1.25 mg/ear) were applied topically into the ear 5 min after TPA. The results of the present study demonstrate that topical BxbRAE-100% strongly inhibited TPA-induced inflammation in a dose-dependent fashion (Figure 4A). The maximum anti-inflammatory effect of the extract (68.2 ± 5.3%) was observed at the dose of 5.7 mg/ear, with an ED_50_ of 1.45 mg/ear. However, indomethacin displayed higher efficacy (89.3 ± 0.9%) and potency than the extract (0.54 mg/ear, Figure 4B).

In the same way the carrageenan-induced paw edema model in mice, a well-validated acute inflammatory model was used to determine the anti-inflammatory activity of BxbRAE-100%. Figure 4C shows that the BxbRAE-100% administrated orally reduced paw edema in a dose-dependent manner at 1, 2, 3, 4, 5, and 6 h after the carrageenan injection in the dose range from 4–400 mg/kg. The groups treated with 0.04 and 0.4 mg/kg of BxbRAE-100% did not show significant changes in paw volume compared to the control group. The reduction of paw edema was observed from the first hour after carrageenan injection, which lasted at least six hours. The maximal reduction in paw edema was observed between 4 and 6 h post carrageenan injection. The analysis of the inhibition of paw edema showed that the oral treatment with BxbRAE-100% inhibited paw edema in a range between 41.75 and 83.16%. The dose of 400 mg/kg most effectively reduced carrageenan-induced paw edema. However, its anti-inflammatory effect was not greater than that of the positive control, indomethacin (Figure 4D). These results showed that BxbRAE-100% produces anti-inflammatory effects whether administered topically or orally.

### 2.4. Inhibition of Proteolytic Activity of BxbRAE-100%

**Figure 5:**
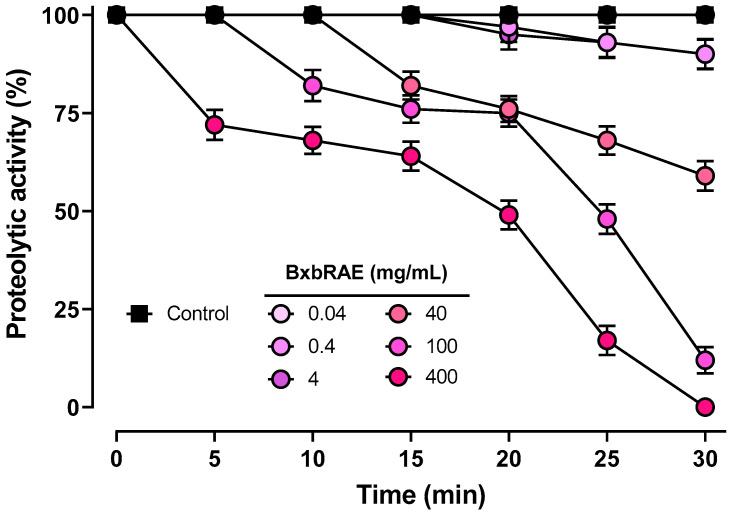
BxbRAE-100% inhibited proteolytic activity in vitro. Each point corresponds to the results of three different preparations.

To determine the anti-inflammatory effect of BxbRAE-100%, the inhibition of proteolytic activity was evaluated using azocasein as a substrate. Figure 5 shows that BxbRAE-100% decreased the proteolytic activity percentage in a concentration- and time-dependent manner. The relative activity percentages induced by the extract after a 30 min incubation period at 40, 100, and 400 mg/mL concentrations were 59%, 12%, and 0%, respectively.

### 2.5. Effect of BxbRAE-100% on the Activity of the Phospholipase A2 and Cyclooxygenase Enzymes

Secretory phospholipase A2 (PLA2) is the first enzyme in the arachidonic acid pathway. This enzyme catalyzes the hydrolysis of membrane phospholipids, releasing arachidonic acid and a lysophospholipid. Subsequently, cyclooxygenase (COX) can oxidize free arachidonic acid to produce prostaglandins, considered important pro-inflammatory mediators [21]. In this study, to preliminarily explore the possible mechanism of action of BxbRAE-100%, the effect of the extract on the activity of sPLA2 and COX enzymes was evaluated. 

#### 2.5.1. Anti-Phospholipase Activity of BxbRAE-100%

**Figure 6:**
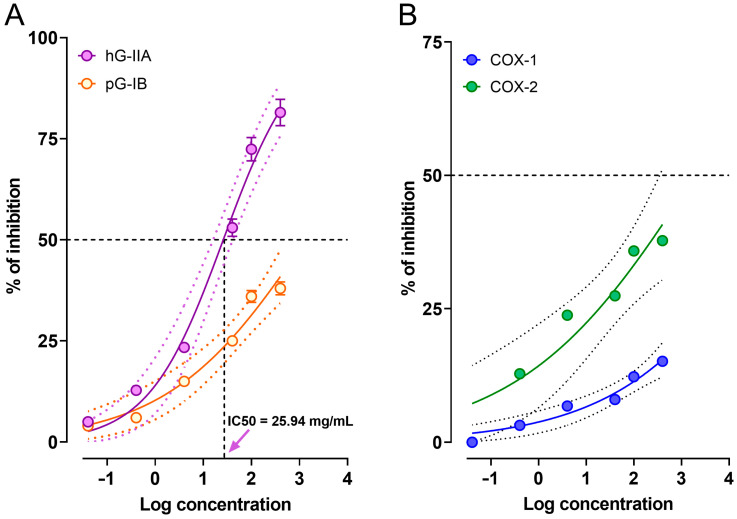
BxbRAE 100% inhibits the activity of sPLA2 and COX isoforms 1 and 2. (**A**) Inhibitory effect of the extract on human group IIA secreted phospholipase A2 (hG-IIA) and porcine group IB phospholipase A2 (pG-IB) activities. (**B**) Inhibitory effect of the extract on the activity of COX-1 and COX-2. The dashed lines represent the concentration that produces 50% inhibition of enzymatic activity (IC_50_). Values represent the average of triplicate measurements.

The extract was assessed for its inhibitory effect toward the two Human group IIA secreted phospholipase A2 (hG-IIA) and Porcine group IB phospholipase A2 (pG-IB) at a range concentration between 0.04 and 400 mg/mL. hG-IIA participates in the inflammatory process, while pG-IB is responsible for the hydrolysis of dietary phospholipids. We hypothesized that the extract could selectively inhibit group IIA pro-inflammatory phospholipase A2, with little or no inhibitory effect on group IB digestive phospholipase A2. The results showed that the extract inhibited in a concentration-dependent manner the activity of hG-IIA. It is important to highlight that the greatest inhibitory activities (72.4 ± 2.9%, and 81.5 ± 3.3%) were observed at the concentrations of 100 and 400 mg/mL. The calculated concentration that inhibits the 50% of hG-IIA activity (IC_50_) was 25.94 mg/mL. On the other hand, the same doses of BxbRAE-100% inhibited pG-IB activity by less than 38% (Figure 6A). These results suggest that inhibition of PLA2 is one of the mechanisms underlying the antinociceptive and anti-inflammatory activities of *B. x buttiana*.

#### 2.5.2. Cyclooxygenase Inhibitory Activity of BxbRAE-100%

In order to evaluate the effect of BxbRAE-100% on the activity of COX-1 and COX-2 isoforms, different concentrations (0.4–400 mg/mL) of BxbRAE-100% were assessed. It was observed that BxbRAE-100% inhibited in a concentration-dependent manner the activity of COX-I and COX-2. The effect was moderate since the highest inhibitory activity was 15.15% and 37.76% for COX-1 and COX-2, respectively. However, as observed, the extract showed higher selectivity for COX-2 than for COX-1 (Figure 6B). Thus, these results suggest that COX inhibition is partially responsible for *B. x buttiana* activities. 

### 2.6. Compounds Identified in BxbRAE 100%

In our previous study, BxbRAE-100% was analyzed via gas chromatography-mass spectrometry (GC-MS). Acetone was used since it allowed for the extraction of secondary plant metabolites of intermediate polarity. Accordingly, the next compounds were identified in the extract: 3-*O*-Methyl-d-glucose, *n*-Hexadecanoic acid, 9,12-Octadecadienoic (Z,Z), 1-Dotriacontanol, Stigmasta-5,22-dien-3-ol and Stigmast-7-en-3-ol, (3β,5α) [19]. Their structure and bioactivities are detailed in Table 1.

### 2.7. In Silico Analysis of the Physicochemical Profile and ADME Properties of BxbRAE-100% Metabolites

At this point, we do not know if just one metabolite or a group of them is responsible for the observed activities of *B. x buttiana*. However, since at least 6 metabolites have been identified in the extract, it is possible to infer that they could be responsible for the observed biological activities. To preliminarily determine whether the compounds found in the acetonic extract of *B. x buttiana* have drug-like properties, an in silico analysis was performed to determine their physicochemical profile and ADME properties using the online free software swissADME, pre-ADME, and pcKSM. The molecular descriptors were calculated according to Lipinski’s rule of five (Ro5), an experimental method to estimate water and lipid solubility, membrane permeability, and pharmacological effectiveness in drug discovery [34]. We found that all six compounds have a molecular weight of less than 500, fewer than five hydrogen bond donors (HBD), and fewer than six hydrogen bond acceptors (HBA). However, they showed, with the exception of 3-*O*-Methyl-d-glucose, a partition coefficient between *n*-octanol and water (log P) greater than five due to their high lipophilicity. These results suggested that the compounds in BxbRAE-100% meet Lipinski’s Ro5 criteria.

Furthermore, the six compounds showed topological polar surface area (TPSA) values lower than 140 Å^2^. Five of them have very small values (37.3 and 23.3), indicating that they can easily cross the cell membrane (Table 2). However, three of the six compounds showed more than 10 rotatable bonds, indicating that they are highly flexible molecules. Altogether, these results suggest that the bioactive compounds present in BxbRAE-100% are highly likely to be absorbed orally and have good cell membrane permeability.

The ADME properties, solubility (log S), human intestinal absorption (HIA), skin penetration (log Kp), and the ability to be a P-glycoprotein substrate (P-gp) were calculated to predict absorption. The results showed that 3-*O*-methyl-d-glucose is highly soluble in water but showed very low HIA and low skin penetration. These properties could affect its bioavailability after oral administration. In contrast, the remaining five compounds were moderately to poorly soluble in water, but their predicted HIA values were greater than 90%. None of them, except 1-Dotriacontanol, are P-gp substrates. Together, these results suggest that the metabolites found in BxbRAE-100% will have moderate to good absorption. For distribution, we predicted descriptors such as plasma protein binding (PPB), brain–blood barrier penetration (BBB), and volume of distribution (Vd). It was found that five of the six compounds showed PPB values very close to 100%, which is consistent with their high log P values, that is, with their lipophilicity.

Furthermore, it was determined that only *n*-Hexadecanoic acid and 9,12-Octadecadienoic acid(Z,Z)- could cross the BBB. All compounds showed Vd values between 0.04–20 L/kg, which are considered optimal values. Finally, 3-*O*-Methyl-d-glucose, *n*-Hexadecanoic acid, 9,12-Octadecadienoic(Z,Z)- and 1-Dotriacontanol showed Cl values < 5 mL/min/kg, indicating low clearance. In contrast, Stigmasta-5,22-dien-3-ol and Stigmast-7-en-3-ol, (3β,5α) showed CL values between 5 and 15 mL/min/kg; suggesting moderate clearance. None of the compounds was a substrate for renal OCT2, suggesting no renal excretion (Table 3).

Drug metabolism is a process that helps to prevent xenobiotics, like drugs, from reaching toxic concentrations and producing toxic side effects. The human cytochrome P450 (CYP) superfamily of enzymes metabolizes both endogenous and exogenous compounds such as drugs, cellular metabolites, and toxins [38]. CYP1A2, 2C9, 2C19, 2D6, and 3A4 isoforms mainly metabolize commercially available drugs. In this study, the metabolism of the compounds present in BxbRAE-100% was analyzed using free online software. The in-silico analysis showed that the six compounds were not CYP1A2, 2C19, 2C9, or 2D6 substrates. Moreover, CYP enzymes also play an essential role in drug–drug and herb–drug interactions. The inhibition of CYP enzymes is strongly associated with adverse drug reactions encompassing metabolic failures and induced side effects. Here we found that *n*-Hexadecanoic acid and 9,12-Octadecadienoic(Z,Z)- are potential inhibitors of CYP1A2. Moreover, *n*-Hexadecanoic acid could also be a CYP2D6 inhibitor. However, it is important to note that most of the compounds did not have the potential of being CYP inhibitors (Table 4). The in-silico results showed that each compound had a unique ADME profile, but, these predictions could help to better understand whether the extract or its metabolites had suitable ADME properties. However, these results need to be rigorously verified through in vitro and in vivo experiments to establish their clinical significance.

## 3. Discussion

In this study, we aimed to expand the knowledge of the biological properties of *Bougainvillea* by evaluating the antinociceptive and anti-inflammatory activities of the acetonic extract of *B. x buttiana* (var. Rose). Our findings showed that BxbRAE-100% significantly reduced nociception in three well-validated murine models of acute pain by acting at peripheral and central levels. 

The acetic acid-induced abdominal constriction model allows the evaluation of the potential analgesic properties of test compounds at the peripheral level since acetic acid stimulates the endings of the primary afferent nerve fibers and produced a localized inflammatory response [39,40,41]. In this model, oral treatment with BxbRAE-100% dose-dependently (0.4 to 400 mg/kg) reduced pain percentages. The groups treated with 40, 100, and 400 mg/kg were more effective in reducing pain than the groups treated with the control drugs aspirin (1 mg/kg), dexamethasone (1 mg/kg), and diclofenac (10 mg/kg). These results suggest that BxbRAE-100% produces analgesic activity at the peripheral level.

Similarly, in the tail immersion test, BxbRAE-100% significantly reduced the pain percentages at 30, 60, and 120 min after oral administration. The efficacy of BxbRAE-100% was comparable to that of the reference drug aspirin but significantly lower than that of dexamethasone. In this test, the opioid drugs, which produce their effects in the central nervous system, show greater sensitivity [41]. Then, our findings suggest an analgesic central action of the extract.

The formalin test is widely used for assessing central and peripheral analgesic activities. This pain model is characterized by a distinctive biphasic nociceptive response. In mice, the early or neurogenic phase occurs between 0 and 5 min after formalin injection into the paw. It is caused by the direct chemical activation of primary nociceptive afferents. The late or inflammatory phase occurs between 15 and 30 min after formalin injection and results from various mechanisms, including inflammation of peripheral tissues and central sensitization [42,43,44]. Our findings showed that BxbRAE-100% reduced, in a dose-dependent manner, the pain percentage in both phases of the formalin test. The most significant effect of BxbRAE-100% was observed at 40, 100, and 400 mg/kg. These doses decreased the percentages of pain in the early and late phases by 70% and 50%, respectively. It is well established that centrally acting drugs, such as opioids, inhibit equally the early and late phases [44], while peripheral acting drugs, such as nonsteroidal anti-inflammatory drugs (NSAIDs; e.g., aspirin) or corticosteroids (e.g., dexamethasone), mainly inhibit the late phase [41]. Thus, our results suggest that BxbRAE-100% has central and peripheral antinociceptive activity in the formalin test. 

The peripheral and central analgesic activity of BxbRAE-100% is consistent with the results of a previous study, which demonstrated that the ethanolic extract of *B. x buttiana* (var. Orange) also produces analgesia in the acetic acid-induced writhing model and the formalin test [16]. 

Several studies have demonstrated the role of inflammation in the development of various health conditions. Inflammation is the body’s response to injury and involves a series of events, including the inflammatory reaction, a sensory response perceived as pain, and the repair process. Infection, tissue injury caused by external noxious stimuli, or immunological injuries are the main causes of the inflammatory response [1,2]. In this study, the anti-inflammatory properties of BxbRAE-100% were determined using both in vivo and in vitro approaches. In vivo, the anti-inflammatory activity was determined with two well-validated models to identify compounds with potential anti-inflammatory activity [45,46]. 

This is the first time the TPA model has been used to test the topically anti-inflammatory properties of *B. x buttiana*. TPA rapidly promotes edema development, peaking 4 to 6 h after topical application and lasting at least 24 h. Our findings show that BxbRAE-100% significantly decreased, in a dose-dependent manner, 4 h post TPA application. The extract achieved its maximum anti-inflammatory effect (68.2 ± 5.3%) with a 5.7 mg/ear dose, and the calculated ED50 was 1.45 mg/ear. However, indomethacin and dexamethasone displayed higher efficacy and potency than the extract. These results confirm the anti-inflammatory effect of BxbRAE-100% and suggest that the topical route could be an alternative to its administration.

The carrageenan-induced paw edema model in rodents is biphasic. The first phase begins immediately after carrageenan injection and decreases within two hours, while the second phase starts at the end of the first phase and remains at least six hours [47]. The mice treated with 4 to 400 mg/kg BxbRAE-100% showed a statistically significant (*p* < 0.001) reduction of carrageenan-induced paw edema in the second phase (between 2 and 6 h) of the inflammatory response. The 400 mg/kg dose produced the maximum edema inhibition (83%). These results were similar to those obtained in the group treated with indomethacin. The second phase in this model is characterized by PGs and bradykinin production, the release of proteases, and the infiltration of neutrophils [47]. Then, the results suggest that the extract produced its anti-inflammatory properties by modulating some of these events. 

It is important to highlight that our findings agree with a previous study that showed the anti-inflammatory properties in vivo and in vitro of *B. x buttiana* (var. Orange) [16,17,18].

Conventional anti-inflammatory treatment includes NSAIDs and corticosteroids, drugs that work by inhibiting elements of inflammatory pathways [7,48]. Corticosteroids have structural similarities to steroid hormones, and one of their mechanisms of action is the inhibition of the pro-inflammatory enzyme phospholipase A2 (PLA2). NSAIDs are structurally diverse drugs that share analgesic, anti-inflammatory, and antipyretic activity. These drugs are classified based on their mechanism of action as non-selective inhibitors or selective COX-2 inhibitors [21,48]. 

In this study, BxbRAE-100% showed activity in two models of inflammatory pain, and in two models of acute inflammation, which are highly sensitive to conventional analgesic and anti-inflammatory treatments. Therefore, we can infer that the pharmacological activities of BxbRAE-100% can be attributed, at least in part, to the inhibition of enzymes involved in the production of pro-inflammatory and pronociceptive mediators. Therefore, to preliminarily explore its mode of action, we evaluated its effect on the activity of PLA2 and COX enzymes.

Until now, ten mammalian subtypes of sPLA2 have been identified: groups IB, IIA, IIC, IID, IIE, IIF, III, V, X, and XII. The second secretory PLA2 (sPLA2), or group II PLA2, is stored in immune cell secretory granules and markedly induced by inflammatory stimuli. Meanwhile, pancreatic sPLA2 IB (pG-IB) mainly participates in the digestion of dietary phospholipids [49]. sPLA2 releases arachidonic acid from membranes, which is the precursor to eicosanoids known for their potent pro-inflammatory actions. In the next step, the rate-limiting reaction enzyme prostaglandin H synthase (PGHS), the peroxidase activity of COX, catalyzes the formation of PGs. The constitutive isoform of COX-1 produces PGs that protect the stomach and kidneys from damage, while the COX-2 isoform is induced during inflammation and produces PGs that play an essential role in pain and inflammation [50]. Therefore, the arachidonic acid pathway has been a target for developing anti-inflammatory treatments.

In this work, our findings showed that the extract inhibited the activity of group IIA PLA2 by 81% with an IC_50_ of 25.94 mg/mL. However, low inhibitory activity was observed for pG-IB. Similarly, we observed that BxbRAE-100% moderately inhibited the activity of COX-1 and COX-2, with a slightly greater selectivity for COX-2. These results suggest that BxbRAE-100% exerts its biological effects by modulating the metabolism of arachidonic acid by reducing the activities of PLA2 and COX enzymes, which decreases the production of pro-inflammatory mediators. 

Protease enzymes found in all living organisms catalyze the cleavage of peptide bonds in other proteins. Human proteases are divided into five mechanistic classes according to the catalytic mechanism of hydrolysis: aspartate, cysteine, metalloproteinases, serine, and threonine proteases [51]. Protease signaling pathways are strictly regulated, and the dysregulation of protease activity can lead to pathologies such as cardiovascular and inflammatory diseases and neurological disorders [52]. In inflammation, rapid protease activity is a key component of the innate immune system that contributes to the microenvironment and regulates the expression and activity of different pro-inflammatory cytokines and for tissue remodeling [53]. Therefore, they are attractive biological target-identifying molecules that can modulate their activity. In this study, BxbRAE-100% significantly decreased the percentage of proteolytic activity in a concentration- and time-dependent manner, reinforcing the evidence of its anti-inflammatory activity. 

Numerous studies have shown the anti-inflammatory activity in plant extracts through in vitro and in vitro studies as well as the identification and characterization of the bioactive compounds involved in their anti-inflammatory action [48,54,55]. This study showed that BxbRAE-100% significantly inhibited edema formation and reduced pain. Therefore, we can infer that the active compounds present in BxbRAE-100%, alone or synergically, are responsible for their anti-inflammatory and analgesic properties. In our previous study, we identified that BxbRAE-100% is composed of the carbohydrate *O*-3-methyl-d-glucose (16.80%); the saturated fatty acid *n*-Hexadecanoic acid (23.10%), the polyunsaturated acid (9-Z,12-Z),-9,12 octadecanoic acid (19.90%), the fatty alcohol 1-Dotriacontanol (19.90%) and the phytosterols (3β,22E)-stigmata-5,22-dien-3-ol (10.60%) and (3β,5α)-stigmast-7-en-3 (9.62%) [19]. These bioactive compounds have been studied and found to produce several pharmacological activities that support their role in the anti-inflammatory properties of *B. x buttiana* var. Rose (see Table 1).

Fatty acids, known for their metabolic role, have now been identified as important regulators of immune function. Among them, *n*-Hexadecanoic acid, also known as Palmitic acid (PA), stands out with its unique properties. This saturated-free fatty acid exhibits antibacterial and antifungal properties [25]. Moreover, this fatty acid also inhibits PLA2, modulates cytokine production, and reprograms the innate immune cell response to secondary inflammatory stimuli in vitro and in vivo [24].

Polyunsaturated fatty acids, essential components in the architecture and function of cell membranes, play a key role in several biological processes, such as metabolic precursors of prostaglandins and leukotrienes [25]. 9,12-Octadecadienoic (Z,Z)-or Linoleic Acid (LA) is the most abundant ω-6 polyunsaturated fatty acid (PUFA) and is essential in the human diet with several functions in health and disease. Several studies have demonstrated that high dietary intake or high plasma concentrations of LA do not appear to result in increased tissue arachidonic acid or in increased in vivo or ex vivo concentrations of inflammatory markers in humans. Moreover, a recent study reported their in vivo and in vitro anti-inflammatory and neuroprotective actions in Parkinson’s disease models [26,27].

Plant-derived sterols, phytosterols, are abundant in lipid-rich plant materials. These compounds are well known for their serum cholesterol-lowering and anti-inflammatory activities [33]. Previous studies found Stigmasta-5,22-dien-3-ol, also called Stigmasterol, in the acetonic extracts of *Sideritis foetens* and the hexane extract of *Eryngium foetidum.* Oral or topical treatment with *Sideritis foetens* extract reduced carrageenan-induced paw edema and inhibited TPA-induced ear edema, respectively. Similarly, the extract of *Eryngium foetidum* inhibited TPA-induced ear inflammation [34,35]. Furthermore, an in vitro study showed that Stigmasterol downregulated PGE2 and MMP-3 in IL-1β-treated cells without affecting IL-6 levels, suggesting that Stigmasterol inhibited the NF-κB pathway [56]. Similarly, in vitro studies showed that Stigmast-7-en-3-ol (3β,5α) inhibited nitric oxide production in macrophages activated by LPS, suggesting potent anti-inflammatory properties [57].

The evidence cited shows that most bioactive compounds reported in BxbRAE-100% exhibit anti-inflammatory activity, suggesting that they could act alone or synergistically to produce the observed analgesic and antinociceptive effects. To better understand how the compounds contribute to the biological activities of the extract, an in silico study was performed to determine the physicochemical profile and ADME properties, as well as the drug-likeness of each compound.

Lipinski’s RO5 was designed to estimate the probability of malabsorption or membrane permeability. If the analysis of a molecule showed that two of the following parameters are out of range, “poor absorption or permeability is possible”: (1) molecular weight greater than 500, (2) more than five hydrogen bond donors, (3) more than 10 hydrogen bond acceptors, and (4) octanol–water partition coefficient (log P) greater than five [58]. The physicochemical properties of all compounds found on BxbRAE-100% met Lipinski´s RO5, suggesting that they will have high oral bioavailability despite their high log P value. These results are consistent with the absorption predictions and the fact that the extract showed efficacy when administered orally. Furthermore, the extract was also active topically, which is consistent with the high lipophilicity of the compounds. The topical activity of BxbRAE-100% opens up the option to investigate different routes of administration that maintain efficacy but limit side effects.

Regarding drug metabolism, we found that five out of six compounds were substrates of CYP3A4. This finding was expected because CYP3A4 is the main isoform involved in more than 50% of the commercially available drugs [59]. Moreover, it was also observed that most of the compounds, except *n*-Hexadecanoic acid and 9,12-Octadecadienoic(Z,Z)-, did not have the potential to be CYP inhibitors. Since inhibition or induction of CYP enzymes may modify the bioavailability or other pharmacokinetic parameters of drugs, which can lead to therapeutic failure and/or toxic effects [60], these results suggest that the BxbRAE-100% had a low potential for herb–drug interactions. While the in silico analysis results are promising, it is crucial that they are rigorously verified through in vitro and in vivo experiments to establish their clinical significance. However, these predictions could significantly enhance our understanding of the ADME properties of the extract or its metabolites.

## 4. Materials and Methods

### 4.1. Chemicals, Drugs and Solvents

Pentobarbital sodium (Lot 20104) was purchased from Laboratorios Aranda S.A de C.V, Querétaro, Mexico. Acetonitrile, azocasein, dexamethasone acetate (Lot LRAC8664), indomethacin (Lot 0000118216), oleic acid, 2-*O*-tetradecanoylphorbol 13-acetate (TPA; Lot MKCL1143), Carrageenan (Lot 095K1394), and bovine pancreas phospholipase A2 were purchased from Sigma-Aldrich Chemical Co. (St. Louis, MO, USA). The COX inhibitor detection assay kit (ovine/human), hG-IIA, and pG-IB phospholipases were purchased from Cayman Chemical Co. (Ann Arbor, MI, USA). Diclofenac was donated by Arroli Organics (Mumbai, India). For the TPA model, the indomethacin, TPA, and the different doses of the extract were dissolved in acetone and applied topically to the ear. For the anti-inflammatory and antinociceptive evaluation, the extract was dissolved in 5% Tween 20 in sterile saline solution and prepared for administration on the day of the experiment.

### 4.2. Animals

In the present study, female mice of the BALB/c strain weighing between 20 and 25 g acquired from the biotherium of the National Institute of Public Health (Cuernavaca-Morelos, Mexico) were used. Before conducting the experiments, the mice were allowed an adaptation period of 7 to 10 days to our laboratory conditions. All experimental procedures followed the Mexican Official Norm of Animal Care and Handling and the International Association for the Study of Pain guidelines. Moreover, the protocol was validated (002/2016) by the Care and Use of Animals of the Faculty of Medicine, UAEM (CCUAL-FM-UAEM). At the end of the experiments, the mice were sacrificed via asphyxiation with CO_2_. All efforts were made to minimize animal suffering and reduce the number of animals used. 

### 4.3. Extraction of Plant Material

The flowers and bracts of *Bougainvillea x buttiana* were collected in Cuernavaca, Morelos, Mexico. A voucher specimen of *Bougainvillea x buttiana* Holttum & Standl was cataloged with the number 33872 for reference and subsequent consultation in the HUMO Herbarium, CIByC (UAEM A mixture of 200 g of flowers and bracts was air-dried for 14 days at 25 °C and ground into powder to obtain the acetonic extract. Nearly 5 g of air-dried powder was poured into 100 mL of 100% acetone (1:20 ratio). This preparation was stored and stirred continuously for 72 h and filtered through Whatman No. 1 filter paper. The residue was subjected to the same procedure three more times. The filtrates obtained on each occasion were mixed and then concentrated at 50 °C, using a rotary evaporator at reduced pressure to produce a dry crude extract. The crude extract was allowed to dry for approximately 72 h at room temperature to determine the percentage yield of the extracts. The extract obtained was called crude acetonic extract of *Bougainvillea x buttiana* (var. Rose) (BxbRAE-100%) and was kept at −20 °C until the experiments were carried out. The BxbRAE-100% performance percentage was calculated using the following formula:

$$\%\;yield=\left(\frac{Weigh\;of\;extract\;material}{Weigh\;of\;original\;plant\;material\;used}\right)\times 100$$

### 4.4. Analgesic Activity

The peripheral antinociceptive activity of BxbRAE-100% was evaluated using the acetic acid-induced writhing model, while the central action was determined using the formalin test and the tail immersion assay.

#### 4.4.1. Acetic Acid-Induced Writhing Model

This test was performed according to the Koster method [61]. Briefly, to evaluate the antinociceptive activity of BxbRAE-100%, 11 independent groups of 4 mice were orally administered with the following treatments: Group 1,vehicle (sterile saline solution 0.9%); Groups 2–7, the BxbRAE-100% at the doses of 0.04, 0.4, 4, 40, 100 and 400 mg/kg, respectively; and Group 8–10 the reference drugs aspirin (1 mg/Kg), diclofenac (10 mg/Kg), and dexamethasone (1 mg/Kg), respectively. Immediately after treatment administration, each mouse was placed in a transparent acrylic box. Sixty minutes later, the mice were injected intraperitoneally with a 0.7% acetic acid solution (0.1 mL/10 g body weight). Then, the number of writhes (contraction of the abdominal muscle together with a stretch of the hind limbs or rotation of the trunk) was immediately quantified 20 min after the intraperitoneal injection of acetic acid [62]. The sum of the number of writhes was used to calculate the percentage of pain (%P) and the % of antinociceptive effect using the following formulas:

$$\%\;Pain=\frac{number\;of\;writhes\;of\;the\;treated\;group}{number\;of\;writhes\;of\;the\;vehicle\;group}\times 100$$

% Antinociceptive effect=100−% Pain

#### 4.4.2. Tail Immersion Assay

This test was carried out following the method described by [63]. This method consists of dipping the lower portion (approximately 4 cm) of the tail of a mouse in hot water (51 ± 1 °C) contained in a one-liter beaker and studying, before and after administration of a potential bioactive substance, the time taken by the animal to remove its tail (latency of tail withdrawal). To determine the antinociceptive effect of the extract, the mice were randomly selected and divided into nine groups of 4 mice each and administered orally, with one of the following treatments: vehicle (physiological saline solution), increasing doses of BxbRAE-100% (0.04, 0.4, 4, 40, 100 and 400 mg/kg) and the positive controls aspirin and dexamethasone (1 mg/kg). Subsequently, the tail withdrawal latency was measured at 0, 30, 60, and 120 min post treatment administration. If the mouse did not withdraw its tail, a cutting time of 10 s was set to avoid tissue damage. The results were expressed as a percentage of pain calculated with the following formula:

% Pain = (Ttcg/Tttg) × 100

where: Tttg = tail withdrawal latency of the treated group and Ttcg = tail withdrawal latency of the vehicle group.

#### 4.4.3. Formalin Test

To determine the antinociceptive effect of the extract, formalin, a model of inflammatory pain, was used following the method described by Rosland et al., 1990 [64]. To apply this model, different groups of 5 mice were treated orally with the following doses of BxbRAE-100% 0.04, 0.4, 4, 40, 100, and 400 mg/kg, the vehicle. Aspirin was used as a positive control (4 mg/kg). Immediately, each mouse was placed in a transparent acrylic cylinder 30 cm in diameter and 30 cm high, surrounded by 30 cm × 30 cm mirrors to observe the mouse throughout the test. One hour after treatment administration, the mouse was removed from the cylinder for subcutaneous injection of 20 μL of 1% formalin into the dorsum of the right hind paw. Immediately thereafter, the mouse was returned to the cylinder, and quantification of nociceptive licking/biting and shuddering behaviors was assessed in two phases. The early phase was evaluated between 0 and 5 min, and the late phase was evaluated between 15 and 30 min after formalin injection.

### 4.5. In Vivo Anti-Inflammatory Activity

#### 4.5.1. TPA-Induced Ear Edema

The mouse model of phorbol 12-myristate 13-acetate (TPA)-induced ear edema was used to determine the topical anti-inflammatory effect of BxbRAE-100% [65]. Briefly, under general anesthesia induced with pentobarbital (63 mg/kg; i.p.), TPA dissolved in ethanol (0.25 μg/μL) was applied to the inner and outer surfaces of the mouse’s ear. Subsequently, the vehicle, increasing doses of the BxbRAE-100% (var. Rosa) (0.1, 0.3, 1, 3, and 5.7 mg/ear) or the positive controls indomethacin (0.25, 0.5, 0.75, 1 and 1.25 mg/ear) and dexamethasone (0.1 mg/ear) were applied in the same way as TPA. Four hours after TPA application, the mice were euthanized via CO_2_ asphyxiation, and a 6-mm ear biopsy was obtained and weighed. The % anti-inflammatory effect was calculated by comparing the biopsies of the TPA groups and the treatments with the TPA + vehicle group. The % anti-inflammatory effect was used to construct the dose–response curve (DRC). Maximum efficacy (maximum anti-inflammatory effect), potency (effective dose 50, ED_50_), and relative potency compared to indomethacin were obtained from the DRC.

#### 4.5.2. Carrageenan-Induced Paw Edema

The carrageenan edema method was carried out according to the method described by Sugishita et al., 1981 [66]. The animals were randomly divided into 8 groups of 5 animals. Six groups were administered orally with different doses of the acetonic extract (0.04, 0.4, 4, 40, 100, and 400 mg/kg); a negative control group did not receive treatment, and the positive control group was treated with 1% indomethacin. One hour after treatment administration, each mouse received an intraplantar injection of 0.1 mL of 1% carrageenan into the right hind paw and into the left paw an equal volume of 0.1% saline solution. The edema produced by carrageenan was measured by using a plethysmograph at different time intervals, and the increase in volume was determined by subtracting the value obtained in the left paw. Next, the area under the curve (AUC) of the time courses was calculated using the trapezoidal rule. The AUC was used to estimate the % Inhibition with the following formula:

$$\%\;of\;inhibition=100-\left(\frac{AUC\;of\;treated\;mice}{UC\;of\;control\;mice}\times 100\right)$$

### 4.6. In Vitro Anti-Inflammatory Activity

#### 4.6.1. Inhibitory Proteolytic Activity of BxbRAE-100%

The activity of BxbRAE-100% was assessed using the colorimetric method previously described by Adulyatham and Owusu-Apenten, 2005 [67] with slight modification. Briefly, 1.0 mg/mL of azocasein as substrate was prepared in 100 mM Potassium phosphate buffer (pH 7). One milliliter of this substrate solution was transferred to an Eppendorf tube containing 0.6 mL of buffer solution and 0.1 mL of different concentrations of BxbRAE-100% (0.04, 0.4, 4, 40, 100, 400 mg/mL). This mixture was then incubated at 60 °C for 20 min. The reaction was stopped by adding 0.3 mL of 10% (*w*/*v*) of trichloroacetic acid solution, followed by centrifugation at 9000× *g* for 10 min at room temperature. The supernatants were collected and mixed with 0.5 mL of 2 M NaOH for 10 min at room temperature. The blank was prepared in the same way as the test samples but with the enzyme previously inactivated in a boiling water bath for 5 min. The final solutions were analyzed spectrophotometrically at an absorbance of 450 nm, and the results were expressed as the percentage of relative activity. All tests were conducted in triplicate (*n* = 3).

#### 4.6.2. Inhibition of Secretory Phospholipase A2 (sPLA2)

The effect of BxbRAE-100% on sPLA2 activity was measured according to the protocol described by de Araújo and Radvanyi, 1987 [68] using inhibitors of group IIA sPLA2, including hG-IIA and pG-IB forms. Briefly, the substrate buffer was prepared with 3.5 mM lecithin solubilized in 100 mM NaCl, 3 mM sodium taurodeoxycholate, 10 mM CaCl_2_, and 0.055 mM phenol red at pH 7.6. Ten microliters of BxbRAE-100% at concentrations of 0.04, 0.4, 4, 40, 100, and 400 mg/mL were mixed with 10 µL of PLA2-GIB and incubated for 20 min at 25 °C. Subsequently, 1 mL of the PLA2 substrate was added to each tube, and the kinetics of hydrolysis were monitored over 5 min by measuring the absorbance at 558 nm. The inhibition percentage was calculated by comparing the residual activity to that of the negative control (absence of BxbRAE-100%). The IC_50_ values were determined from the resulting inhibition curves.

#### 4.6.3. Cyclooxygenase Inhibitory Activity

The in vitro COX-1/2 inhibitory activity of BxbRAE-100% was evaluated using a COX (Ovine/Human) inhibitor screening kit with 96-well plates purchased from Cayman Chemical (Ann Arbor, MI, USA). The assay was performed according to the manufacturer´s instructions. This screening measures PGF2 produced by SnCl_2_ reduction of COX-derived PGH2. Initial COX-2 activity was measured using 105 µL of reaction buffer and 10 µL of COX-1/2 enzymes. Similarly, COX-1/2 inhibitor tubes were prepared by adding 20 µL of each concentration of BxbRAE in each tube in addition to the above ingredients. The background tubes correspond to inactivated COX-1/2 enzymes by boiling water for 3 min. For the initial reactions, 10 µL of arachidonic acid was added to each tube and quenched with 50 µL of 1 M HCl. The PGH2 formed was reduced to PGF2 a by adding 100 µL SnCl_2_ and reading the plate at 405 nm. The results were expressed as the percentage of inhibition.

### 4.7. In Silico Analysis of Compounds Present in BxbRAE-100%

The molecular descriptors and drug likeliness of the compounds identified in BxbRAE-100% were analyzed according to Lipinski’s RO5 (Table 5). The parameters to predict the properties of Absorption, Distribution, Metabolism, and Elimination (ADME) (Table 5) were calculated with the three widely used online free software: SwissADME (http://www.swissadme.ch/ accessed on 31 May 2024), PreADMET 2.0 (https://preadmet.webservice.bmdrc.org/ accessed on 31 May 2024), and pkCSM (https://biosig.lab.uq.edu.au/pkcsm/prediction accessed on 31 May 2024) [69,70]. In all the cases, the descriptors calculation was conducted by uploading the standard smile files on the web servers.

### 4.8. Statistical Analysis

Data are expressed as the mean ± SD. Statistical analysis was conducted using GraphPad Prism 8 (GraphPad Software, Inc., San Diego, CA, USA). One-way ANOVA followed by a post hoc test of Dunnet was used to determine statistical significance between more than three experimental groups. The significance level was set at *p* < 0.05.

## 5. Conclusions

BxbRAE showed significant antinociceptive and anti-inflammatory activities in vivo and in vitro, at least in part through the inhibition of PLA2 and COX. These activities can be attributed to the unique or synergistic action of the bioactive compounds present in the extract. These compounds meet the Lipinski Ro5 criteria, suggesting good bioavailability for oral administration. These findings support the use of *Bougainvillea x buttiana* in traditional Mexican medicine. Furthermore, they highlight the potential of *B. x buttiana* extracts to continue studying them in order to develop new phytomedicines for pain and inflammation treatment.

## Figures and Tables

**Table 1:** Compounds identified in BxbRAE-100% and their reported bioactivities.

Compound Name and Structure	Activity	Reference
**Carbohydrates**		
3-*O*-Methyl-d-glucose 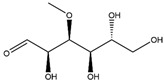	Increased the rate of hexose transport into myotubes by increasing glucose transporter type 4 (GLUT-4) activity	[22,23]
**Saturated Fatty Acids**		
*n*-Hexadecanoic acid 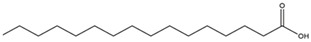	Antioxidant, anti-inflammatory, antinociceptive, hypocholesterolemic, nematicide, hemolytic, anti-androgenic, 5-alfa reductase inhibitor and immunomodulator	[22,23,24,25]
**Polyunsaturated Fatty Acids**		
9,12-Octadecadienoic(Z,Z)- 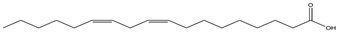	Antioxidant, antibacterial, anti-inflammatory neuroprotective, hypocholesterolemic, cancer preventive, hepatoprotector, antihistaminic, and antieczemic	[26,27,28,29,30]
**Fatty Alcohol**		
1-Dotriacontanol 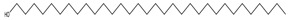	Antimicrobial, and antioxidant	[31,32]
**Phytosterols**		
Stigmasta-5,22-dien-3-ol 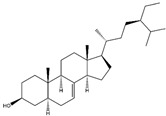	Antioxidant, anti-inflammatory, antibacterial, cytotoxic, anti-hepatotoxic, antiviral, and hypercholesteremic	[33,34,35]
Stigmast-7-en-3-ol, (3β,5α) 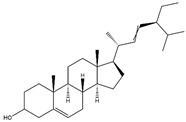	Anti-cancer, antidiabetic, and anti-inflammatory	[36,37]

**Table 2:** Physicochemical profiles of compounds found in BxbRAE-100% according to Lipinski’s Ro5.

Compound	MW	nHBD	nHBA	Log P	N_Vio_	Meet Lipinski RO5 Criteria	TPSA (Å^2^)	nRotB
	<500	<5	<10	≤5	<1	Yes/No	<140	<10
3-*O*-Methyl-d-glucose	194.18	4	6	−2.25	0	Yes	107.22	6
*n*-Hexadecanoic acid	256.43	1	2	7.06	1	Yes	37.30	14
9,12-Octadecadienoic(Z,Z)-	280.45	1	2	6.86	1	Yes	37.30	13
1-Dotriacontanol	466.88	1	1	10.11	1	Yes	20.23	30
Stigmasta-5,22-dien-3-ol	414.72	1	1	8.62	1	Yes	20.23	6
Stigmast-7-en-3-ol, (3β,5α)	414.72	1	1	8.05	1	Yes	20.23	5

MW: molecular weight; nHBD: number of hydrogen-bond donors; nHBA: number of hydrogen bond acceptors; Log P: logarithm of partition coefficient of the compound between *n*-octanol and water; N_Vio_: number of RO5 violated; TPSA: topological polar surface area; nRotB: Num. rotatable bonds.

**Table 3:** ADME properties of the bioactive compounds identified on BxbRAE-100%.

Compound	Absorption				Distribution			Excretion	
	Log S	HIA	Log Kp	P-gp	PPB%	BBB	Vd	Cl_tot_	Renal OCT2 Substrate
3-*O*-Methyl-d-glucose	1.17 Highly soluble	27.2	−9.54	No	29.67	No	0.43	1.84	No
*n*-Hexadecanoic acid	−5.02 Moderately soluble	91.9	−2.77	No	98.95	Yes	0.61	2.38	No
9,12-Octadecadienoic (Z,Z)-	−4.67 Moderately soluble	90.4	−3.37	No	98.95	Yes	0.61	2.37	No
1-Dotriacontanol	−10.7 Insoluble	84.3	−2.06	Yes	100	No	5.1	4.73	No
Stigmasta-5,22-dien-3-ol	−7.74 Poorly soluble	95.9	−2.38	No	95.04	No	1.27	7.35	No
Stigmast-7-en-3-ol, (3β,5α)	−7.99 Poorly soluble	97.8	−2.17	No	95.17	No	1.46	11.31	No

Water solubility: Log S (mol/L); human intestinal absorption: HIA (%); human skin permeability: Log Kp (cm/h); plasma protein binding: PPB (%); blood–brain barrier permeability: BBB (Log BB); volume of distribution: Vd; total clearance: Cl_tot_ (Log mL/min/kg); organic cation transporter 2: OCT2.

**Table 4:** Metabolism profile predicted in silico.

Compound	Metabolism									
	CYP1A2		CYP2C9		CYP2C19		CYP2D6		CYP3A4	
	S	I	S	I	S	I	S	I	S	I
3-*O*-Methyl-d-glucose	No	No	No	No	No	No	No	No	No	No
*n*-Hexadecanoic acid	No	**Yes**	No	No	No	No	No	**Yes**	**Yes**	No
9,12-Octadecadienoic(Z,Z)-	No	**Yes**	No	**Yes**	No	No	No	No	**Yes**	No
1-Dotriacontanol	No	No	No	No	No	No	No	No	**Yes**	No
Stigmasta-5,22-dien-3-ol	No	No	No	No	No	No	No	No	**Yes**	No
Stigmast-7-en-3-ol, (3β,5α)	No	No	No	No	No	No	No	No	**Yes**	No

S: substrate; I: inhibitor.

**Table 5:** Molecular descriptors calculated in silico.

Physicochemical profile	Lipinski’s RO5 descriptors	Molecular weight (MW) Number of hydrogen bond donors (nHBD) Number of hydrogen bond acceptors (nHBA) Partition coefficient of the compound between *n*-octanol and water (log P) Topological polar surface area (TPSA) Num. of rotatable bonds (nRotB)
ADME properties	Absorption	Aqueous solubility in mol/L (Log S) Human intestinal absorption (%HIA) Skin permeation coefficient (LogKp) P-glycoprotein substrate(P-gp)
	Distribution	Plasma protein binding (%PPB) Blood–Brain Barrier permeability (BBB) Volume of distribution (Vd)
	Metabolism	Phase I of drug metabolism: Cytochrome P450 isoenzyme: CYP1A2 CYP2C9; CYP2C19, CYP2D6 CYP3A4
	Excretion	Total body clearance (Cltot) Renal organic cation transporter 2 (OCT2) Substrate

## Data Availability

The original contributions presented in the study are included in the article, further inquiries can be directed to the corresponding author.
